# *In Vitro* Storage of Functional Sperm at Room Temperature in Zebrafish and Medaka

**DOI:** 10.1089/zeb.2023.0054

**Published:** 2023-12-14

**Authors:** Kazumasa Takemoto, Toshiya Nishimura, Toshihiro Kawasaki, Yukiko Imai, Karine Levy, Neta Hart, Ivan Olaya, Sean M. Burgess, Yaniv M. Elkouby, Minoru Tanaka, Noriyoshi Sakai

**Affiliations:** ^1^ Department of Genetics, School of Life Science, SOKENDAI (The Graduate University for Advanced Studies), Mishima, Japan.; ^2^ Division of Biological Science, Nagoya University, Nagoya, Japan.; ^3^ Department of Gene Function and Phenomics, National Institute of Genetics, Mishima, Japan.; ^4^ Deparment of Developmental Biology and Cancer Research, Hebrew University of Jerusalem Faculty of Medicine, Jerusalem, Israel.; ^5^ Institute for Medical Research Israel-Canada (IMRIC), Jerusalem, Israel.; ^6^ Department of Molecular and Cellular Biology, University of California, Davis, Davis, California, USA.; ^7^ Integrative Genetics and Genomics Graduate Group, University of California, Davis, Davis, California, USA.

**Keywords:** sperm storage, lactic acid, zebrafish, medaka

## Abstract

The longevity of sperm in teleost such as zebrafish and medaka is short when isolated even in saline-balanced solution at a physiological temperature. In contrast, some internal fertilizers exhibit the long-term storage of sperm, >10 months, in the female reproductive tract. This evidence implies that sperm in teleost possesses the ability to survive for a long time under suitable conditions; however, these conditions are not well understood. In this study, we show that the sperm of zebrafish can survive and maintain fertility in L-15-based storage medium supplemented with bovine serum albumin, fetal bovine serum, glucose, and lactic acid for 28 days at room temperature. The fertilized embryos developed to normal fertile adults. This storage medium was effective in medaka sperm stored for 7 days at room temperature. These results suggest that sperm from external fertilizer zebrafish and medaka has the ability to survive for at least 4 and 1 week, respectively, in the body fluid-like medium at a physiological temperature. This sperm storage method allows researchers to ship sperm by low-cost methods and to investigate key factors for motility and fertile ability in those sperm.

## Introduction

Spermatozoa are one of the most differentiated cells. This cell has unique features of a small head comprising a nucleus and a flagellum with minimal cytoplasm and gene expression. There are reports that sperm in some teleosts can be stored for short term in saline-balanced solution at a cold temperature.^1^ Temperatures <6°C reduce sperm metabolism and bacterial growth and are generally favored over storage at higher temperature. However, although the motility of guppy (*Poecilia reticulata*) sperm is maintained for up to 120 min in Hank's balanced salt solution (HBSS) at room temperature,^2^ female guppies can store sperm in the reproductive tract beyond lifetime of the donated male.^3^

The long-term storage of sperm in the female reproductive tract and the spermatheca is found in many animals of internal fertilizers such as mollusks, insects, teleost, amphibians, reptiles, birds, and mammals.^4–6^ This evidence implies that sperm has the ability to survive for long periods of time at the physiological temperature under appropriate conditions that are similar to the microenvironment of the female reproductive tract and the spermatheca of these organisms. The microenvironment that supports long-term survival is not yet well understood.

Zebrafish is an external fertilizer. Sperm held in ice-cold HBSS can fertilize eggs efficiently for up to 90 min.^7^ However, we have observed that *in vitro* differentiated sperm can typically survive 3–4 days under zebrafish spermatogonia culture.^8,9^ Moreover, these cells start to swim normally after being transferred to hypotonic water. In this study, we addressed the establishment of *in vitro* storage conditions of zebrafish sperm based on the conditions that support spermatogonia culture.

We succeeded in storing fertile zebrafish sperm for 28 days at room temperature by supplementation of L-15 medium with bovine serum albumin (BSA), fetal bovine serum (FBS), glucose (Glu), and lactic acid (LA). Furthermore, we found that this medium was effective in storing sperm of another external fertilizer medaka, which is phylogenetically distant from zebrafish.

## Materials and Methods

### Animals

Zebrafish of the India strain (provided by Dr. Y. Kishimoto, National Institute of Genetics, Japan), the AB strain (provided by Dr. U. Strähle, Karlsruher Institute of Technology, Germany) or *vas::EGFP* transgenic lines^10^ (provided by Dr. LC. Olsen, Bergen High Technology Centre, Norway) were used. Medaka of *OKcab* (provided by Dr. K. Naruse, National Institute for Basic Biology, Japan) was used. The experiments were conducted in accordance with the guidelines of the National Institute of Genetics, Nagoya University, the Hebrew University, and UC Davis.

### Collection, maintenance, and artificial fertilization using zebrafish sperm

Male zebrafish were anesthetized with 0.01% ethyl *p*-aminobenzoate (Wako, Osaka, Japan). After wiping with ethanol cotton, the sperm was sucked into a pipette tip from urogenital opening by gentle abdominal massage and suspended in the L-15-based storage medium. The storage medium was made from L-15 (314 mOsm/kgH_2_O; Sigma) by the addition of the following stock solutions: 1/100 of 5000 U/mL penicillin, 5000 μg/mL streptomycin (Gibco), 1/80 of 2 M Glu (Wako), 3/100 of FBS (Biowest), 1/10 of 5% BSA (w/v, fraction V; Sigma), 1/10 of Milli-Q water, 1/100 of 1 M Hepes (pH 7.9 adjusted by NaOH; Wako), 1/10,000 of 1 M LA (Sigma), and 1/10,000 of 1 M reduced glutathione (GSH; Sigma) to the total volume. Glu, BSA, Hepes, LA and GSH were dissolved in Milli-Q water, and filtered at 0.2 μm. Milli Q water was autoclaved.

Finally, the storage medium contained 50 U/mL penicillin, 50 μg/mL streptomycin, 25 mM Glu, 3% FBS, 0.5% (w/v) BSA, 10 mM Hepes (pH 7.9), 22% Milli Q water, and 0.1 mM LA or 0.1 mM GSH, and was 291–292 mOsm/kgH_2_O (*n* = 2) using Osmometer (Vogel). The number of sperm was counted using a hemocytometer and stored at concentration of 37,000, 74,000, or 148,000 sperm cells/μL in 40 μL in 1.5 mL tubes at 23°C or 4°C.

Oocytes (unfertilized eggs) were prepared from wild-type females according to the method of Westerfield.^7^ In all experiments, 100–200 oocytes were used for each fertilization. Considering individual differences in oocyte quality, a single female was squeezed two times (or three times as shown in Fig. 2C, D), successively, to release oocytes on separate plates. The same batch of oocytes was used to evaluate each pair (or series) of sperm samples. The sperm suspension was added to the dish, which was then shaken gently for 2 min to mix. PBS (100 μL) was added gradually, with continued shaking. After an additional 2 min, 5 mL of fish water was gradually added to each plate. Successful fertilization was assessed at 5–6 h postfertilization by examination using a stereomicroscope.

### Collection, maintenance, and artificial fertilization using medaka sperm

A male medaka fish was anesthetized with 0.01% ethyl *p*-aminobenzoate. Testes were removed and transferred to a balanced salt solution (BSS) containing 0.5% bleach for 30 s, and washed twice with BSS. Sperm were collected by tearing the testis with fine forceps in 66 μL of sperm storage medium. The zebrafish sperm storage medium was modified for medaka to contain 0.2 mM LA. The number of sperm was counted using a hemocytometer and then stored at 450,000 sperm cells/mL in 100 μL in 1.5 mL tubes at 23°C or 4°C. Oocytes were collected from 8 to 12 females and pooled. The cells were divided into four groups (10–20 oocytes in each) and used for each series of experimental conditions in duplicate. Artificial fertilization was performed as described previously.^11^

### Genotyping

PCR was performed with genomic DNAs extracted from sperm and embryos at 7 days postfertilization (dpf). PCR was performed with GoTaq Green Master Mix (Promega) using specific primers to the *EGFP* sequence (up:accacatgaagcagcacgact, dn:cttctcgttggggtctttgc) and the genomic *spo11* locus.^12^ PCR products were examined by agarose gel electrophoresis.

### Statistical analysis

Data are presented as the mean ± standard deviation of at least four independent experiments. Statistical differences between the target comparison groups were determined using Welch's *t*-test. Results of statistical test were indicated as *(*p* ≤ 0.05), **(*p* ≤ 0.01), ***(*p* ≤ 0.001), or ****(*p* ≤ 0.0001). *p* ≤ 0.05 was considered statistically significant.

## Results

### L-15 medium supplemented with FBS, BSA, and Glu prolonged the fertilization ability of zebrafish sperm

L-15 medium is established for culture in free gas exchange with the atmosphere,^13^ and is used for the culture of zebrafish spermatogonia to functional sperm as the basal medium.^8^ Therefore, we used L-15 medium for zebrafish sperm storage. Since zebrafish sperm has been maintained in ice-cold HBSS,^7^ we initially stored sperm at 4°C. After testing different concentrations of sperm, 1.5 × 10^6^, 3.0 × 10^6^, and 6.0 × 10^6^ cells/40 μL (Supplementary Fig. S1), we found that 3.0 × 10^6^ cells/40 μL was optimal for the fertilization assay. The addition of 50 U/mL penicillin and 50 μg/mL streptomycin to L-15 medium did not decrease fertility after storage for 4 days (Supplementary Fig. S2).

**FIG. 1.**
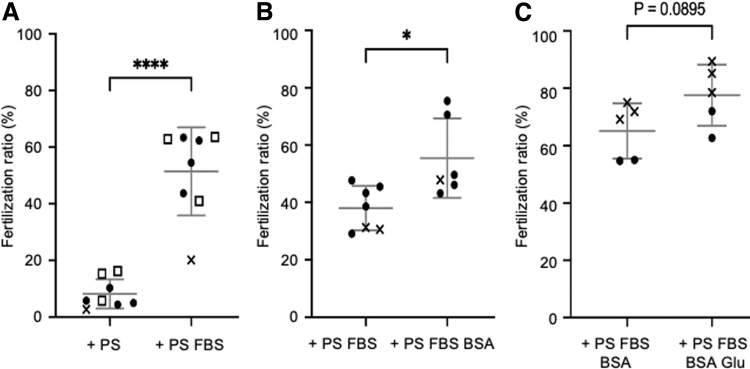
Effect of FBS, BSA, and Glu on the fertility of stored sperm. Pooled sperm from several males were stored in L-15 containing PS with/without FBS, BSA, and Glu at 4°C for 4 days, and used to fertilize 100–200 oocytes. **(A)** Effect of FBS (*n* = 8). **(B)** Effect of BSA (*n* = 6–7). **(C)** Effect of Glu (*n* = 5). The shape of the marks indicates the same sampling batch of pooled sperm. The mean was calculated by combining all results. Error bars indicate the standard deviation. *P ≤ 0.05, ****P ≤ 0.0001. BSA, bovine serum albumin; FBS, fetal bovine serum; Glu, glucose; PS, penicillin/streptomycin.

We first examined the effect of FBS and BSA that are used for the culture of zebrafish spermatogonia,^8,9^ and found that 3% FBS increased the fertility of stored sperm six times higher (51.5% ± 15.6%) than did the medium without FBS for 4 days at 4°C (8.2% ± 5.1%; Fig. 1A). BSA at 0.5% (w/v) also increased sperm fertility significantly (from 38.0% ± 7.7% to 55.5% ± 13.8%) after 4 days of storage at 4°C (Fig. 1B). In addition, glycolysis plays a major role in adenosine triphosphate supplementation in mouse sperm flagellar movement.^14^ The addition of 25 mM Glu resulted in a slight increase in the fertility of stored sperm at 4°C, but this effect was not significant (Fig. 1C).

### Effects of antioxidants, LA and GSH, on the maintenance of zebrafish sperm

Since LA is reported as a sperm motility inactivation factor in the sperm storage tubules in birds,^15^ its effect on zebrafish sperm storage was examined. The addition of 0.1 mM LA decreased the fertility after 4 days of storage at 4°C (Supplementary Fig. S3). Although the sperm survived, we observed that the motility of sperm stored in the LA-containing medium was weaker than that in medium without LA after the induction by the addition of fresh water. We speculated that the combination of LA and low temperature at 4°C excessively suppressed sperm motility.

**FIG. 2.**
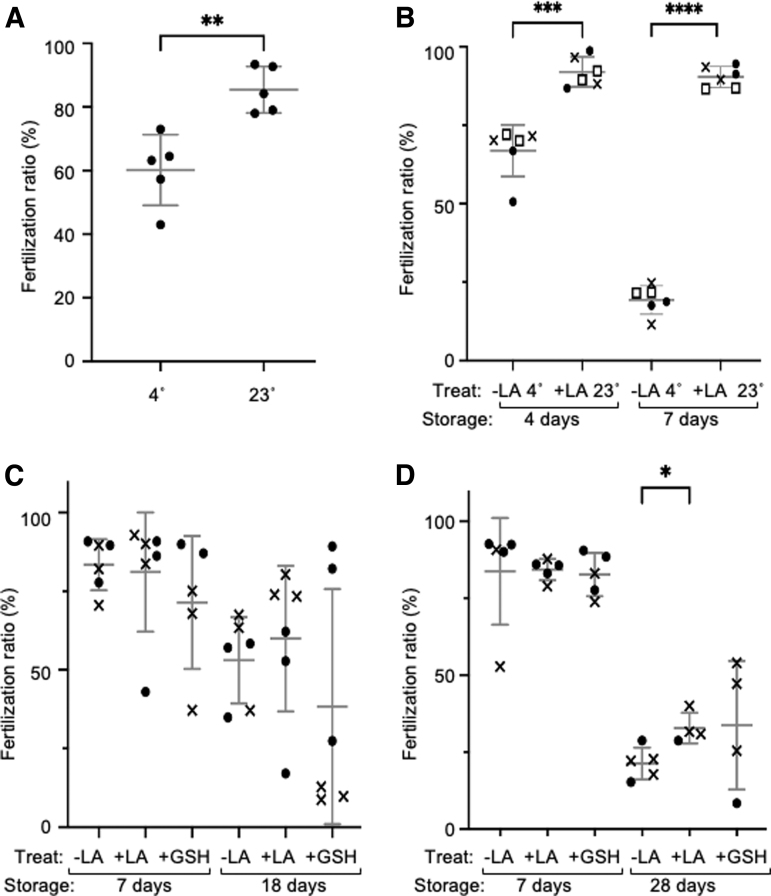
Effect of LA, GSH, and the storage temperature on the fertility of stored sperm. Pooled sperm from several males were stored in L-15 containing PS, FBS, BSA, Glu with/without LA or GSH, and used to fertilize 100–200 oocytes. **(A)** Comparison of stored sperm at 4°C and 23°C in the LA-containing medium for 4 days (*n* = 5). **(B)** Comparison of stored sperm at 4°C without LA and 23°C with LA for 4 and 7 days (*n* = 6). **(C, D)** Effect of LA and GSH on the stored sperm for 18 days **(C,** *n* = 5–6**)** and 28 days **(D,** *n* = 4–5**)** at 23°C. The shape of the marks indicates the same sampling batch of pooled sperm. The mean was calculated by combining all results. Error bars indicate the standard deviation. *P ≤ 0.05, **P ≤ 0.01, ***P ≤ 0.001, ****P ≤ 0.0001. GSH, glutathione; LA, lactic acid.

Thus, we examined different storage temperatures. When sperm was stored at 23°C, the fertility was significantly increased compared with that of sperm stored at 4°C, in LA-containing medium (Fig. 2A). Interestingly, when comparing sperm stored at 4°C without LA with those stored at 23°C with LA, the latter sperm showed higher fertilization ability at 92.5% ± 4.8% and 92.2% ± 3.3% than the former sperm at 66.9% ± 8.2% and 19.3% ± 4.5% after 4 and 7 days of storage, respectively (Fig. 2B). These results indicate that LA-containing medium at room temperature is suitable for the long-term storage of zebrafish sperm.

It has been shown previously that the fertility of sperm produced by *in vitro* culture at a low O_2_ concentration is greater than that produced at a normal O_2_ concentration.^9^ Since LA has a reductive property, we reasoned that it might stimulate low O_2_ conditions. In addition, antioxidant GSH has been shown to enhance the fertility of frozen/thawed mouse sperm.^16^ Therefore, we compared the effect of LA and GSH on fertility. After 18 days, sperm stored in the 0.1 mM LA-containing medium showed slightly higher fertilization rates at 70.0% ± 23.2% than did those stored in GSH at 28.3% ± 37.3% after 18 days (Fig. 2C).

**FIG. 3.**
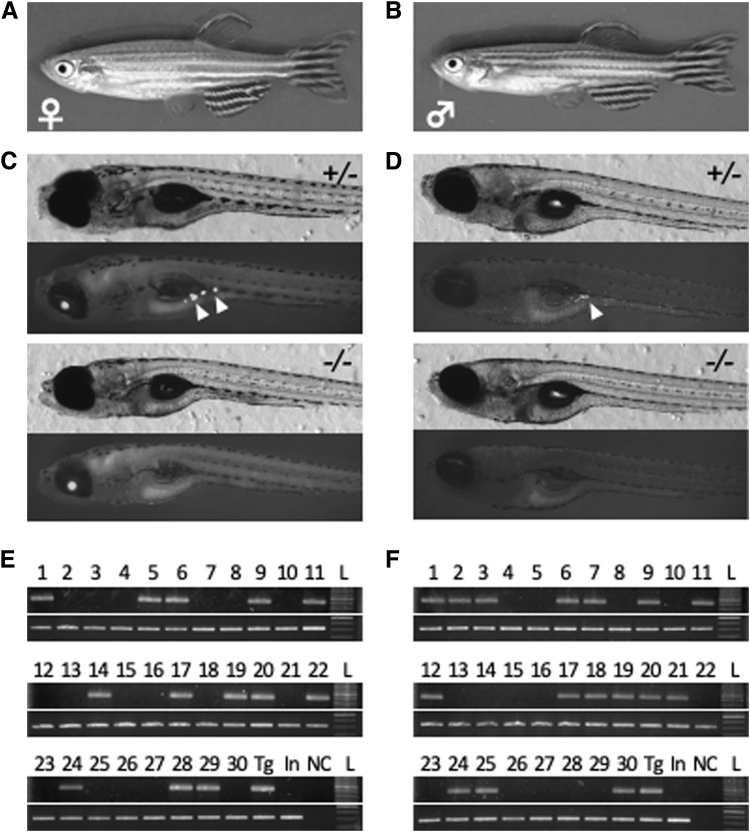
Development of fertilized embryos from stored sperm for 28 days. **(A, B)** Adult female **(A)** and male **(B)** fish (F1) that were obtained by *in vitro* fertilization of stored *vas::EGFP* sperm to wild-type oocytes. **(C, D)** Transmission of the *EGFP* gene to the next generation. EGFP expression in gonads was detected in almost half of the next generation (F2) at 12 dpf from F1 female **(C)** and F1 male **(D)** mating with the wild-type. *Upper panels* indicate heterozygous (+/−) EGFP-positive gonads (*arrow heads*), and *lower panels* indicate EGFP-negative (−/−) gonads. **(E, F)** Genomic PCR analysis of the *EGFP* gene in the next generation. The gene was transmitted in almost half of 30 progenies (F2) from F1 female **(E)** and F1 male **(F)** mating with the wild-type. *Upper panels* indicate amplification of *EGFP* and *lower panels* indicate amplification of genomic *spo11* locus. Genomic DNAs collected from sperm of the *vas::EGFP* line (Tg) and wild-type (In: India) were used as controls. NC: negative control with water. L: ladder. dpf, days post fertilization.

Variation of fertility outcomes in the presence of GSH was high, so we decided it would not be a reliable supplement. Sperm stored at 23°C with LA exhibited fertility 32.8% ± 5.0% after 28 days of storage (Fig. 2D). The fertilized embryos developed normally and spawned as adults. When sperm of the *vas::EGFP* transgenic line^10^ was fertilized after 28 days of storage, the fluorescence of the EGFP protein was confirmed to be maintained and transmitted to the germ line (Fig. 3).

These results suggest that zebrafish sperm has a property to survive for long term *in vitro* when stored in an L-15-based medium supplemented with FBS, BSA, Glu, and LA as a reducing agent. As a trial, zebrafish sperm were transported in storage medium between Jerusalem, Israel, and Mishima, Japan, and from Mishima to Davis California, United States.

When 2–3 × 10^6^ sperm cells/40 μL was put in a 0.5 mL tube and transported in an envelope at the ambient temperature, embryos at the 49.5%–82.1% fertilization rate was obtained after 10 days at the Sakai laboratory in Mishima, at 12.5%–16.8% after 6 days at the Elkouby laboratory located in Jerusalem, and at 21.2% after 5 days at the Burgess laboratory located in Davis (Supplementary Table S1). Thus, we show that zebrafish sperm can be shipped internationally without dry-ice or ice packs and maintain sufficient levels of fertility.

### Sperm storage medium prolongs the fertilization ability of medaka sperm

The medium based on L-15 and supplemented with FBS, BSA, Glu, and LA as described above maintained the fertility of zebrafish sperm for as long as 28 days. Thus, we examined whether this storage medium can be applied to the sperm of another external fertilizing fish, medaka. It is reported that medaka sperm motility was 14%–17% in HBSS after 7 days at 4°C.^17^

**FIG. 4.**
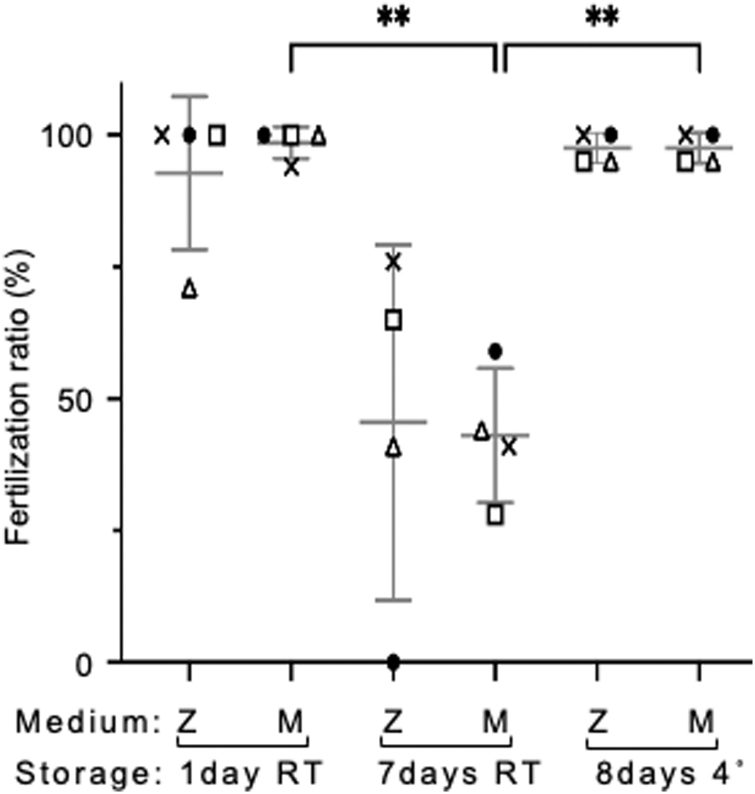
Storage of medaka sperm in L-15 containing FBS, BSA, Glu, and LA. Sperm from individual males were divided and stored in the zebrafish medium (Z) (L-15 containing FBS, BSA, Glu, and LA) and the medaka medium (M, same components of the zebrafish medium, except twofold of LA) at 4°C or 23°C. The shape of the marks indicates the same sampling batch of sperm. The mean was calculated by combining all results (*n* = 4). Error bars indicate the standard deviation. **P ≤ 0.01.

When medaka sperm was stored in the zebrafish sperm storage medium for 7 days at 23°C and for 8 days at 4°C, fertilized eggs were successfully obtained by the artificial fertilization at 45.5% ± 29.2% and 97.5% ± 2.5%, respectively (Fig. 4). The fertility of sperm stored in LA at 4°C was not decreased compared with that of those stored in LA at 23°C, unlike zebrafish sperm. This result is presumably because medaka is a temperate zone fish. We sometimes observed spoiled storage medium as showing as yellow (a pH decrease) at 23°C, and sperm stored in the spoiled storage medium resulted in low fertility.

Since medaka sperm were collected by tearing the testis with fine forceps in the sperm storage medium, we speculated that testicular somatic cells dissociated during tearing grew excessively. Therefore, we modified the zebrafish medium by increasing LA twofold to create the medaka storage medium. The medaka medium maintained the fertility of medaka sperm at 43.0% ± 11.0% for 7 days at 23°C and 97.5% ± 2.5% for 8 days at 4°C, which was almost equivalent to the fertility of sperm stored in zebrafish sperm storage medium, but with decreased variation (Fig. 4). These results indicate that medaka sperm can be stored *in vitro* in medium similar to zebrafish sperm storage medium at room temperature for at least 1 week, although storage at a low temperature is likely more suitable for long-term storage.

## Discussion

This study shows that the sperm of zebrafish and phylogenetic distant medaka can be stored *in vitro* under a novel composition of the storage medium that contains FBS and BSA, which were used for the spermatogonia culture system^8,9^ at room temperature. Furthermore, the addition of LA increased fertilization rates. LA has been reported to be a potential antioxidant.^18^ The antioxidant GSH was also effective on sperm preservation, but less so than LA. One reason may be that GSH is toxic to zebrafish sperm that led to high variation of outcomes.

Oxidative stress can lead to sperm damage, deformity, and eventually male infertility in humans.^19^ Furthermore, near-anoxia induces immobilization and sustains viability of sperm stored in ant queens.^20^ These pieces of evidence provide the possibility that avoidance of oxidative stress is one of the important factors for sperm survival.

Among the phylogenetic distance between zebrafish and medaka, most species in teleost exist.^21^ We predict this storage method may be extended for use in other teleost fishes. The medaka storage medium was not as effective as the zebrafish storage medium, however, varying the concentrations of supplements could improve storage efficiency. Several different orders of internal fertilizing teleosts, such as guppy, Shiner perch (*Cymatogaster aggregata*), *Helicolenus dactylopterus*, and *Sebastes schlegeli*, store sperm in the female reproductive tract for long periods of time.^22–24^ It would be interesting to further evaluate the composition of fluids in the female reproductive tract of these teleosts. Comparative analysis of the fluid composition will lead to improvements in storage medium that allow longer term sperm storage as well as a better understanding of how sperm can be stored for long periods of time in nature.

Since sperm is a transcriptionally silent cell, motility depends on the activation and/or inhibition of key signaling pathways.^25^ However, the complete sperm motility process is far from fully known. Since a vast number of sperm are easily prepared, a number of chemicals can be screened simultaneously. The media and assays described here can be used to screen chemicals that affect sperm motility and fertility, which facilitate understanding key mechanisms that secure sperm motility and fertility. In addition, the storage of their sperm for >7 days at ambient temperature makes it possible to ship mutant and transgenic lines without dry-ice and at dramatically lower costs of transportation compared with embryos or adult fish.

This new method of sperm storage will greatly facility the addition of lines to stock centers and accelerate research using mutant and transgenic lines. It should be noted that at this time, we cannot rule out unforeseen problematic outcomes of sperm storage, such as DNA damage due to oxidative stress. Thus in the short term, it is advisable to use the method with some degree of caution.

## Supplementary Material

Supplemental data

Supplemental data

Supplemental data

Supplemental data

## Data Availability

All data generated or analyzed during this study are included in this published article and its Supplementary Information files.
