# Supplementary material for: In Vitro Storage of Functional Sperm at Room Temperature in Zebrafish and Medaka
Source: Zebrafish. 2023 Dec 14;20(6):229–35. doi: 10.1089/zeb.2023.0054 (PMC11075172; doi:10.1089/zeb.2023.0054)
Supplement: Supplemental data [file Suppl_FigureS1.docx]

**
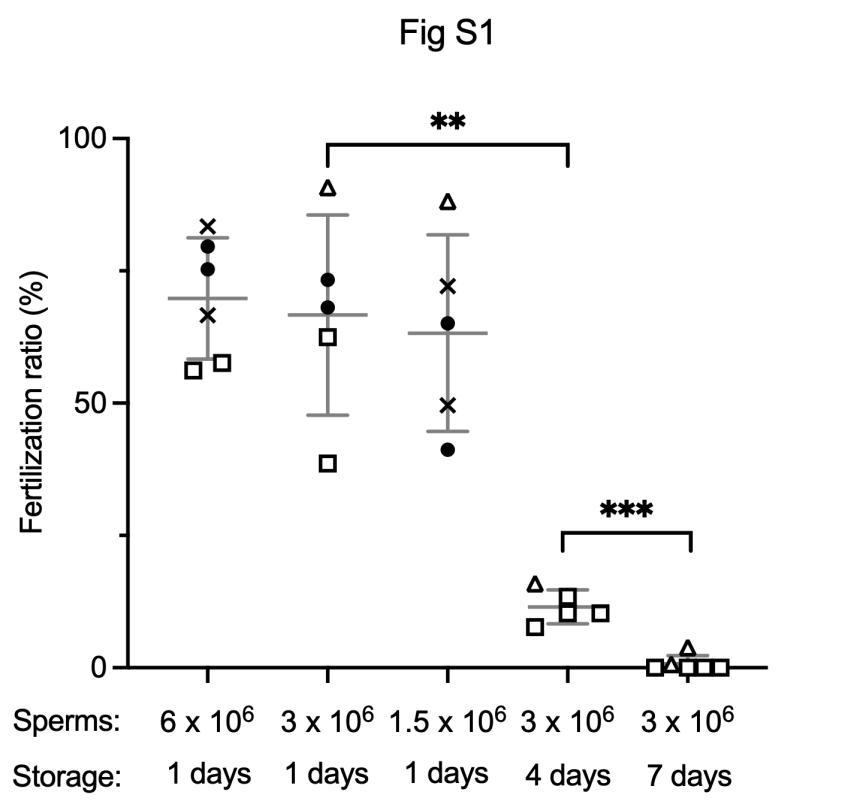
**

**Supplemental Fig. S1.** Assessment of the number of sperm to fertilize oocytes. Sperm was pooled from several males, suspended in L-15 medium, stored in the refrigerator (4˚C), and used to fertilize 100-200 oocytes. The shape of the marks indicates the same sampling batch of pooled sperm. The mean was calculated by combining all results (n=5-6). Error bars indicate the standard deviation.
