# Supplementary material for: In Vitro Storage of Functional Sperm at Room Temperature in Zebrafish and Medaka
Source: Zebrafish. 2023 Dec 14;20(6):229–35. doi: 10.1089/zeb.2023.0054 (PMC11075172; doi:10.1089/zeb.2023.0054)
Supplement: Supplemental data [file Suppl_FigureS2.docx]

**
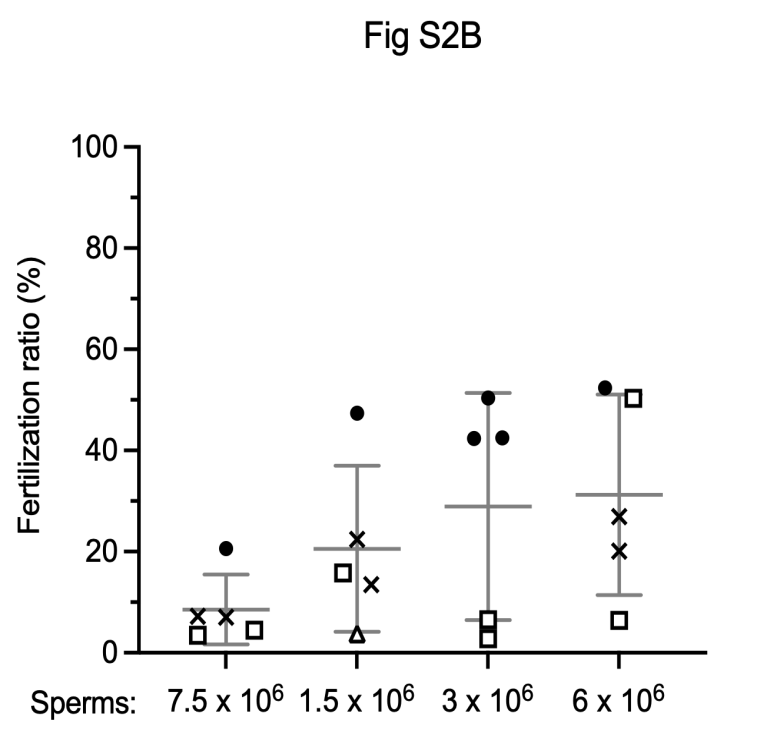
A B**

**
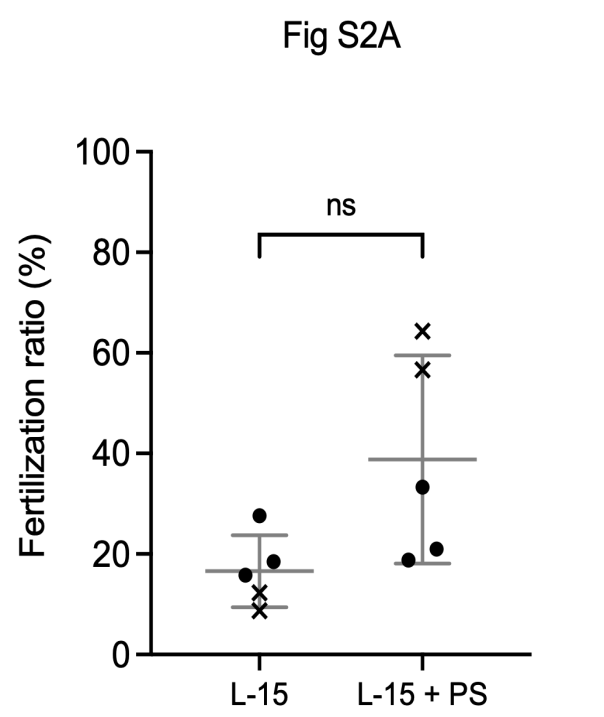
**

**Supplemental Fig. S2.** Effect of penicillin/streptomycin on the fertility of stored sperm. Pooled sperm from several males was stored in L-15 with/without penicillin/streptomycin (PS) at 4˚C for 4 days, and used to fertilize 100-200 oocytes. **A** Effect of PS. The same batch of oocytes from a single female was used for each pair of medium conditions. **B** Different numbers of sperm were stored in L-15 with PS. The shape of the marks indicates the same sampling batch of pooled sperm. The mean was calculated by combining all results (n=5). Error bars indicate the standard deviation.
