# Supplementary material for: In Vitro Storage of Functional Sperm at Room Temperature in Zebrafish and Medaka
Source: Zebrafish. 2023 Dec 14;20(6):229–35. doi: 10.1089/zeb.2023.0054 (PMC11075172; doi:10.1089/zeb.2023.0054)
Supplement: Supplemental data [file Suppl_FigureS3.docx]

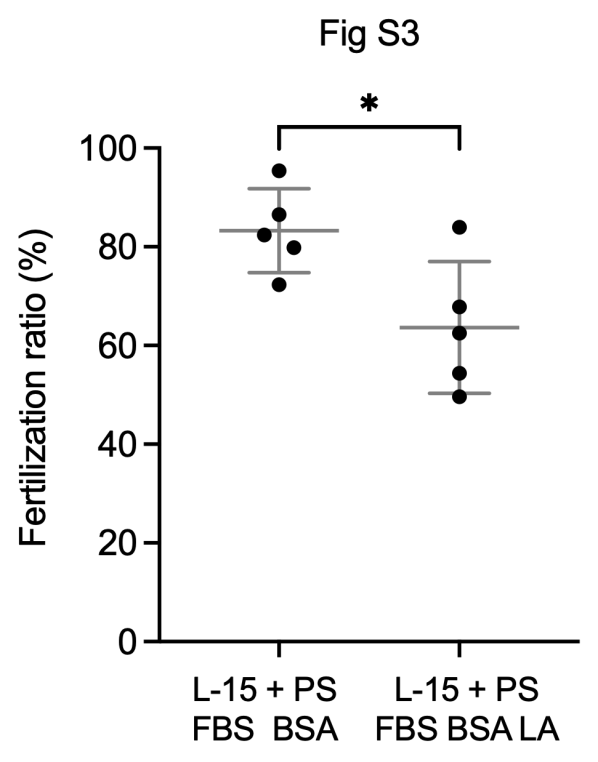


**Supplemental Fig. S3.** Effect of lactic acid (LA) on the stored sperm at 4˚C. Pooled sperm from several males was stored in in L-15 containing penicillin/streptomycin (PS), FBS, BSA with/without LA for 4 days. The shape of the marks indicates the same sampling batch of pooled sperm. The mean was calculated by combining all results (n=5). Error bars indicate the standard deviation.
