# Supplementary material for: In Vitro Storage of Functional Sperm at Room Temperature in Zebrafish and Medaka
Source: Zebrafish. 2023 Dec 14;20(6):229–35. doi: 10.1089/zeb.2023.0054 (PMC11075172; doi:10.1089/zeb.2023.0054)
Supplement: Supplemental data [file Suppl_TableS1.docx]

**Supplementary Information**

**Supplemental Table S1.** Fertilization rate of sperm transported at an ambient temperature between Mishima and Jerusalem or from Mishima to Davis California.

A. From Jerusalem to Mishima

| Sample # | Days kept | Vol. sperm (μl) | Rep. | Egg quality | # total eggs | # fertilized eggs | Fertilization rate (%) |
| --- | --- | --- | --- | --- | --- | --- | --- |
| No. 1 | 10 | 15 | 1 | good | 78 | 64 | 82.1 |
| No. 1 | 10 | 15 | 2 | good | 73 | 41 | 56.2 |
| No. 2 | 10 | 15 | 1 | good | 113 | 66 | 58.4 |
| No. 2 | 10 | 15 | 2 | good | 97 | 48 | 49.5 |
| No. 3 | 10 | 15 | 1 | good | 79 | 41 | 51.9 |
| No. 3 | 10 | 15 | 2 | good | 82 | 42 | 51.2 |

B. From Mishima to Jerusalem

| Sample # | Days kept | Vol. sperm (μl) | Rep. | Egg quality | # total eggs | # fertilized eggs | Fertilization rate (%) |
| --- | --- | --- | --- | --- | --- | --- | --- |
| No. 4 | 6 | 15 | 1 | good | 250 | 42 | 16.8 |
| No. 5 | 6 | 15 | 1 | good | 143 | 18 | 12.6 |

C. From Mishima to Davis California

| Sample # | Days kept | Vol. sperm (μl) | Rep. | Egg quality | # total eggs | # fertilized eggs | Fertilization rate (%) |
| --- | --- | --- | --- | --- | --- | --- | --- |
| No. 6 | 5 | 40 | 1 | good | 608* | 129* | 21.2 |
| No. 6 | 5 | 40 | 2 | good |  |  |  |

*Both were pooled and counted.
